# Feasibility of the Use of Combinatorial Chemokine Arrays to Study Blood and CSF in Multiple Sclerosis

**DOI:** 10.1371/journal.pone.0081007

**Published:** 2013-11-20

**Authors:** Keith R. Edwards, Jaya Goyal, Tatiana Plavina, Julie Czerkowicz, Susan Goelz, Ann Ranger, Diego Cadavid, Jeffrey L. Browning

**Affiliations:** 1 Multiple Sclerosis Center of NE New York, Latham, New York, United States of America; 2 Department of Neurology, Beth Israel Deaconess Medical Center, Boston, Massachusetts, United States of America; 3 Department of Translational Medicine, Biogen Idec, Cambridge, Massachusetts, United States of America; 4 Department of Immunobiology, Biogen Idec, Cambridge, Massachusetts, United States of America; 5 Department of Neurology Clinical Development, Biogen Idec, Cambridge, Massachusetts, United States of America; Institute Biomedical Research August Pi Sunyer (IDIBAPS) - Hospital Clinic of Barcelona, Spain

## Abstract

Meningeal inflammation, including the presence of semi-organized tertiary lymphoid tissue, has been associated with cortical pathology at autopsy in secondary progressive multiple sclerosis (SPMS).  Accessible and robust biochemical markers of cortical inflammation for use in SPMS clinical trials are needed.  Increased levels of chemokines in the cerebrospinal fluid (CSF) can report on inflammatory processes occurring in the cerebral cortex of MS patients.  A multiplexed chemokine array that included BAFF, a high sensitivity CXCL13 assay and composite chemokine scores were developed to explore differences in lymphoid (CXCL12, CXCL13, CCL19 and CCL21) and inflammatory (CCL2, CXCL9, CXCL10 and CXCL11) chemokines in a small pilot study.  Paired CSF and serum samples were obtained from healthy controls (n=12), relapsing-remitting MS (RRMS) (n=21) and SPMS (N=12). A subset of the RRMS patients (n = 9) was assessed upon disease exacerbation and 1 month later following iv methylprednisone. SPMS patients were sampled twice to ascertain stability. Both lymphoid and inflammatory chemokines were elevated in RRMS and SPMS with the highest levels found in the active RRMS group. Inflammatory and lymphoid chemokine signatures were defined and generally correlated with each other. This small exploratory clinical study shows the feasibility of measuring complex and potentially more robust chemokine signatures in the CSF of MS patients during clinical trials. No differences were found between stable RRMS and SPMS. Future trials with larger patient cohorts with this chemokine array are needed to further characterize the differences, or the lack thereof, between stable RRMS and SPMS.

## Introduction

The pathology that drives progressive forms of MS remains poorly understood. SPMS was viewed traditionally as primarily a neurodegenerative process, yet recent studies indicate that neurodegeneration in SPMS is secondary to inflammation [1–4]. Extensive analysis of chronic MS autopsy brains from the UK MS tissue bank revealed the presence of meningeal based inflammation and ectopic or tertiary lymphoid tissue (TLT) in a large number of SPMS subjects [3]. Analysis of brain biopsies collected at an early stage of MS revealed the presence of cortical lesions with active demyelination in 38% of the patients [5]. The TLT found in the meninges of a large percentage of SPMS subjects contained proliferating B cells, specialized stromal reticular cell networks, segregated T and B cell areas, and production of the B cell chemokine CXCL13 [6,7]. More recently, PET imaging of microglial activation in the MS cerebral cortex was found to be associated with disability [8].

It remains unclear if there are fundamentally different inflammatory pathologies leading to the various lesions in the subpial and cortical gray matter relative to the white matter in MS as well as what is the relative contribution of the cortical component to progressive MS. Likewise, it is uncertain whether the TLT-type CNS events are particularly pathogenic by providing nurturing and stable B-cell niches in the meninges surrounding the brain and spinal cord, although a recent rodent EAE study suggested TLT were critical for determinant spreading in T cells [9–12]. 

In MS, inflammation is associated with altered patterns of chemokine and cytokine expression [13]. In general, levels of a wide range of chemokines are elevated in inflammation, however, a subset is expressed constitutively in the lymphoid organs, often termed “lymphoid” chemokines [14,15]. In the normal state, these lymphoid chemokines serve to orchestrate lymphocyte trafficking into lymphatics, across the vasculature into the lymph node parenchyma as well as to position lymphocyte subsets selectively in proximity to specialized stromal cell networks. Notably, these lymphoid chemokines are increased in chronic inflammatory settings where they can drive the formation of more organized lymphoid microenvironments and probably also affect less organized events [14]. The levels of several lymphoid chemokines are elevated in the CSF of MS patients and the B cell positioning chemokine, CXCL13, in particular, has been well-studied [13]. CXCL13 levels in the CSF increase at the onset of MS in clinically isolated syndrome (CIS), increase further with exacerbation in RRMS, subside at an older age, and have been associated with disease progression and higher risk genotypes [16–27]. Increased expression of other lymphoid chemokines such CCL19 and CXCL12 have also been described in these settings [23,28,29]. 

It is important to understand if RRMS and SPMS have different chemokine signatures in either CSF or the blood. Robust analytical methods are needed to measure longitudinal changes in the levels of these chemokines that can be present at rather low levels. In this manuscript, we explore the utility of quantitative panels of inflammatory chemokines, i.e. CCL2, CXCL9, CXCL10 and CXCL11 as well as lymphoid chemokines, CXCL12, CXCL13, CCL19 and CCL21, for use in blood and CSF. The methodology was rigorously validated, and an additional high sensitivity assay was developed for CXCL13 in CSF to adequately address the shortcoming of the existing method. 

## Patients and Methods

### Study subjects

Serum and CSF samples were collected at the Multiple Sclerosis Center of Northeastern New York (Latham, NY) from 12 clinically stable RRMS (RRMS) patients who did not have any relapses or steroid treatment for at least 60 days; nine RRMS patients with an acute exacerbation by Schumacher’s criteria (EX-RRMS); 12 SPMS patients diagnosed on the basis of accumulation of disability independently of relapses and without evidence of ongoing relapses and/or steroid treatment for at least 90 days; and 12 healthy volunteers as normal controls (NC). All nine EX-RRMS subjects were seen within 48 hours of calling the MS Center with new onset symptoms of fewer than 6 days and clinically evaluated for objective findings. They underwent lumbar puncture (LP) the same day with blood drawn within 20 minutes of the LP. The first dose of 1000 mg of IVMP was begun within one hour of the LP for the first of three consecutive days. Eight of 9 EX-RRMS subjects received a brain MRI within 24 hours of the LP. One month after IVMP, these patients had a second LP. SPMS subjects underwent a second LP within 1-18 months after the first LP. Blood and CSF were obtained from MS patients with approval of the BioMed Institutional Review Board for the Multiple Sclerosis Center of NE New York. All patients signed informed consent.

### Serum and CSF collection

When lumbar punctures were performed, venous blood samples were collected into serum separator tubes. Following centrifugation, the serum was stored frozen at -80°C until analysis. The CSF was centrifuged immediately at 200g for 10 minutes at 2-8°C, and the supernatant was removed, aliquotted into Eppendorf tubes and immediately stored at -80°C. Contamination of the CSF by blood was carefully assessed by multiple criteria including gross appearance, RBC counts, presence of hemoglobin or Defensin-A3 (neutrophil) RNA transcripts and Affymetrix hemoglobin and neutrophil RNA signatures (Ann Ranger, unpublished data). 

### Analytical Assays

All chemokine assays including CXCL13 immuno PCR (i-PCR), and multiplex assays were qualified using fit-for-purpose biomarker assay qualification to assure assay performance [30]. In preliminary evaluations, individual CXCL13 and CCL19 ELISA kits were used (R&D Systems). Serum concentration of albumin was measured using the Olympus Albumin method, which measures absorbance of the albumin-bromocresol green complex bichromatically (600/800 nm).  CSF concentration of albumin was measured using nephelometry on an Olympus analyzer. IgG (E88-104), IgA (E88-102), and IgM (E88-100) in both serum and CSF were quantitated using specific ELISAs (Bethyl Laboratories, Montgomery, TX).  An ELISA-based determination may minimize the potential complication of various oligomeric states of IgA and IgM when using nephelometric methods. The Ig index was calculated by correcting the ratio of CSF to serum values (also referred to as the Q value) with the CSF and serum albumin values according to standard methodology. 

#### Immuno PCR (iPCR)

Monoclonal anti-human CXCL13 antibody (*R&D Systems MAb 801*) conjugated to DNA (mAb-DNA) was prepared by chemical cross-linking, and purified by preparative chromatograpy (Chimera Biotech, Imperacer® proprietary method). Capture antibody (anti-human CXCL13, R&D Systems) was coated onto TopYield modules (Nunc), blocked to minimize non-specific binding, and then incubated overnight at 4° C with 1:1 mixture of CSF sample and the DNA-tagged anti-CXCL13 mAb. After incubation, modules were washed and PCR master mix added to each well. The modules were sealed and placed in a real-time PCR cycler. The resulting delta Ct values were plotted against CXCL13 concentration. A standard curve was constructed from the obtained delta Ct values using a four-parameter curve fit model and the concentration of CXCL13 in each sample was extrapolated from the curve. 

#### Chemokine Multiplex Assay

A custom multiplex assay consisting of the analytes CXCL9, CXCL10, CXCL11, CXCL12, CXCL13, CCL2, CCL19, CCL21, and BAFF was developed for both human serum and CSF matrices using the Luminex/XMAP platform. For each analyte, a panel of antibodies was conjugated to beads (capture reagent) and biotin (detection reagent). Each capture:detection antibody pair was screened by a single Luminex immunoassay using recombinant protein as the standard and human serum or CSF as assay matrix. The final antibody pairs were selected based on the sensitivity, specificity, and the ability to detect individual analytes at biologically relevant levels. Performance of each assay was then further optimized as a part of a multiplex to ensure minimal cross reactivity while maintaining the sensitivity toward individual analytes. Blinded samples (serum n=24, CSF n=54) previously tested in individual ELISAs were analyzed in the multiplex assay to determine the correlation of the results obtained in the multiplex assay compared to those obtained in each individual ELISA. The lower limit of quantification (LLOQ) for all the chemokines tested in serum and CSF matrices are shown in Table 1. 

**Table 1:** Lower limit of quantitation of chemokines and BAFF in pg/ml by multiplex ELISA in serum and CSF.

Analyte	Serum	CSF
CXCL9	91	151
CXCL10	51	29
CXCL11	35	13
CXCL12	40	31
CXCL13	28	22
CXCL13 (iPCR)	ND	1
CCL2	21	23
CCL19	63	26
CCL21	10	10
BAFF	15	13

### Data Analysis

Normalized CSF composite chemokine scores were created as described [31]. In short, values were normalized between 0 and 1 such that the 95^th^ percentile of all patient data was the upper limit and levels higher than that were assigned the value 1. With a composite score derived from three chemokines, the maximum value was 3. All composite scores were then transformed to a 100 point scale. With the serum chemokine data, there was little movement and hence a composite scale as described above was inappropriate. Therefore, individual chemokine levels were converted to z-values and the composite scores represent the mean of the individual z-values. Statistical analyses were performed using one-way ANOVA (Kruskal-Wallis with a Dunn’s post test and p<0.05 was considered significant. Correlations between individual chemokine levels, Q_Ig_ ratios, etc were assessed using the Spearman’s rank test. Arms on the box and whisker plots show the 10-90% range in all figures. 

## Results

### Study subjects

A total of 12 normal controls and 33 MS subjects from a single center were enrolled into the study and the baseline characteristics are defined in Table 2. Disease duration was similar in the stable RRMS and SPMS groups and shorter in the EX-RRMS group. Most MS subjects were disabled with a mean Expanded Disability Status Scale (EDSS) score of 4.4 in the RRMS groups and 6.6 in the SPMS group and an average Timed 25-Foot Walk of 8.4 seconds in the RRMS groups and 11.2 seconds in the SPMS group. Relapses in the previous year were at least 5 times more frequent in the RRMS groups than in the SPMS group and the median time from conversion from RRMS for the SPMS cohort was 8.7 years. Six of 8 Ex-RRMS patients had one or more gadolinium-enhancing lesions on brain MRI obtained within 24 hours of the initial CSF sampling and initiation of IVMP treatment. Brain MRI activity was not assessed in one EX-RRMS subject. Most MS subjects (both RRMS and SPMS) were on treatment or had received treatment recently with MS disease modifying agents, including immunomodulatory and chemotherapeutic treatments (Table 2). Notably, 7 patients were receiving interferon-ß (IFN-ß), 3 RRMS, 1 EX-RRMS and 3 SPMS. 

**Table 2:** Baseline characteristics of the MS patients and healthy controls.

	RRMS-EX	RRMS-STABLE	SPMS	Normal Controls
Number of Subjects	9	12	12	12
Mean (SD) age in years	41 (5)	46 (8)	46 (5)	38 (9)
Gender Female (%)	6 (67%)	8 (67%)	9 (82%)	5 (62.5%)
MS Duration (median years)	5.12	13.5	13.0^1^	N/A^2^
Mean (SD) relapses prior year	1.7 (0.76)	1.1 (1.38)	0.2 (0.4)	N/A
Mean EDSS score (SD)	4.3 (1.06)	4.5 (1.64)	6.5 (0.47)	N/A
Mean (SD) Timed 25 Foot Walk	8.9 (3.4)	8.4 (5.6)	11.2 (2.73)	N/A
Percentage without walking aid (n)	78% (7)	75% (9)	9% (1)	N/A
Percentage with Gd+ lesions**^*3*^**	75% (6/8)	N/A	N/A	N/A
Q Albumin	4.49	4.06	4.31	3.16
Igg Index	0.88	0.84	1.02	0.11
Oligoclonal bands (mean)	7.44	6.75	4.54	0.0
*Current MS Treatment (# patients)*				
ABR**^*4*^**	1	3	3	N/A
Natalizumab	4	3	0	N/A
Cyclophosphamide	0	1	6	N/A
Other**^*5*^**	0	4	2	N/A
None	4	1	3	N/A

^1^ Median interval from conversion from RRMS to SPMS was 8.7 years.

^2^ N/A not available

^3^ Gd+, gadolinium-enhancing lesions

^4^ Interferon, i.e. Avonex®, Betaseron® or Rebif®

^5^ Other includes Copaxone®, mycophenylate, teriflunomide and IVIG

### Quantitation of Serum and CSF Chemokine Levels

**Figure 1:**
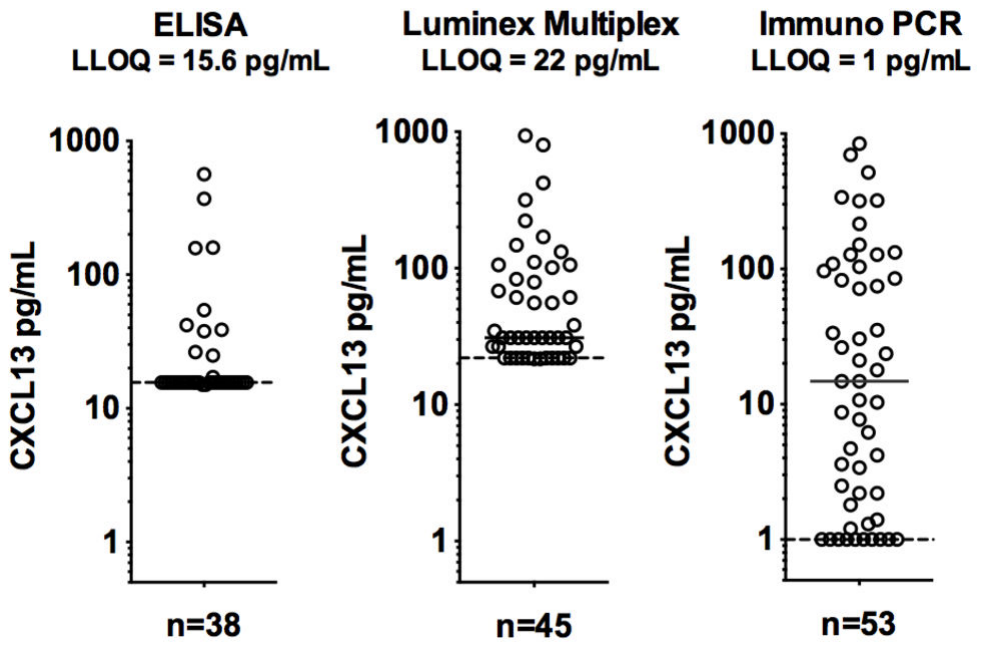
Comparison of three different assays to quantitate CXCL13 in CSF. “ELISA” refers to a commercial kit (R&D), the “Luminex” is a custom chemokine multiplex assay and Immuno-PCR refers to the ELISA with PCR based quantitation. Samples from all four cohorts of patients are included. The low limit of quantitation (LLOQ) was determined for CXCL13 in CSF and is not the buffer-based assay performance.

 The standard commercial R&D ELISA for CXCL13 was found to be limiting in sensitivity for many CSF samples. The minimal detectable level of CXCL13 in buffer was 1-2 pg/mL; however, the CXCL13 level in CSF that can be quantified with acceptable precision and accuracy was determined to be 15 pg/ml based on the spike-recovery of CXCL13 into a panel of individual CSF matrices. Therefore reporting values below 15 pg/mL in CSF may be unreliable. To quantitate low levels of CXCL13 in CSF, two additional assays were explored, a Luminex multiplex array (LLOQ = 22 pg/ml in CSF) and a highly sensitive iPCR assay with excellent sensitivity and performance with CSF samples (LLOQ =1 pg/mL). For CSF samples with measurable CXCL13 concentrations by ELISA (>15.6 pg/mL), an excellent correlation was observed between the ELISA and the iPCR ELISA (r^2^=0.89); however, the iPCR assay was able to measure CXCL13 concentrations in 81% (43 out of 53) of all CSF samples tested as compared to only 29% (11 out of 38) with the commercial ELISA (Figure 1). We therefore used CXCL13 values from the multiplex assay to evaluate the serum samples and the iPCR assay for CSF samples. The Luminex multiplex was used to quantitate the remaining chemokines and BAFF in both serum and CSF. 

The range of chemokine concentrations was consistent with those from published studies e.g. with baseline values from a study of rituximab efficacy in MS [32]. The lower CSF CXCL13 levels in SPMS when compared to EX-RRMS were comparable to a recent study [25]. Serum CCL21 levels were similar to previous reports, yet in contrast to our results, CSF CCL21 levels were reported as undetectable in two studies [23,28]. In another study, CSF CCL21 levels appeared to be roughly in the range we observed [29]. The observed difference may be due to the assays/reagents utilized for the measurements. In our experience and consistent with Krumbholz et al [28], CSF CCL21 levels were undetectable using R&D Systems ELISA assay, whereas our multiplex assay and the system of Pashenkov et al, where CSF CC21was quantifiable, both used biotinylated polyclonal goat anti-human CCL21 antibody (R&D Systems AF366) [29]. Secondly, serum CXCL9 levels were about 10 fold higher than typically reported although CXCL9 and CXCL10 correlated well indicating that the values were proportionally accurate [33]. CSF CXCL9 levels appeared similar to published values [34]. Both serum and CSF CXCL10 levels were slightly higher than those reported earlier [35], and CSF CXCL10 levels were lower than reported in another study [36]. 

### Lymphoid vs Inflammatory Chemokines in Serum and CSF

**Figure 2:**
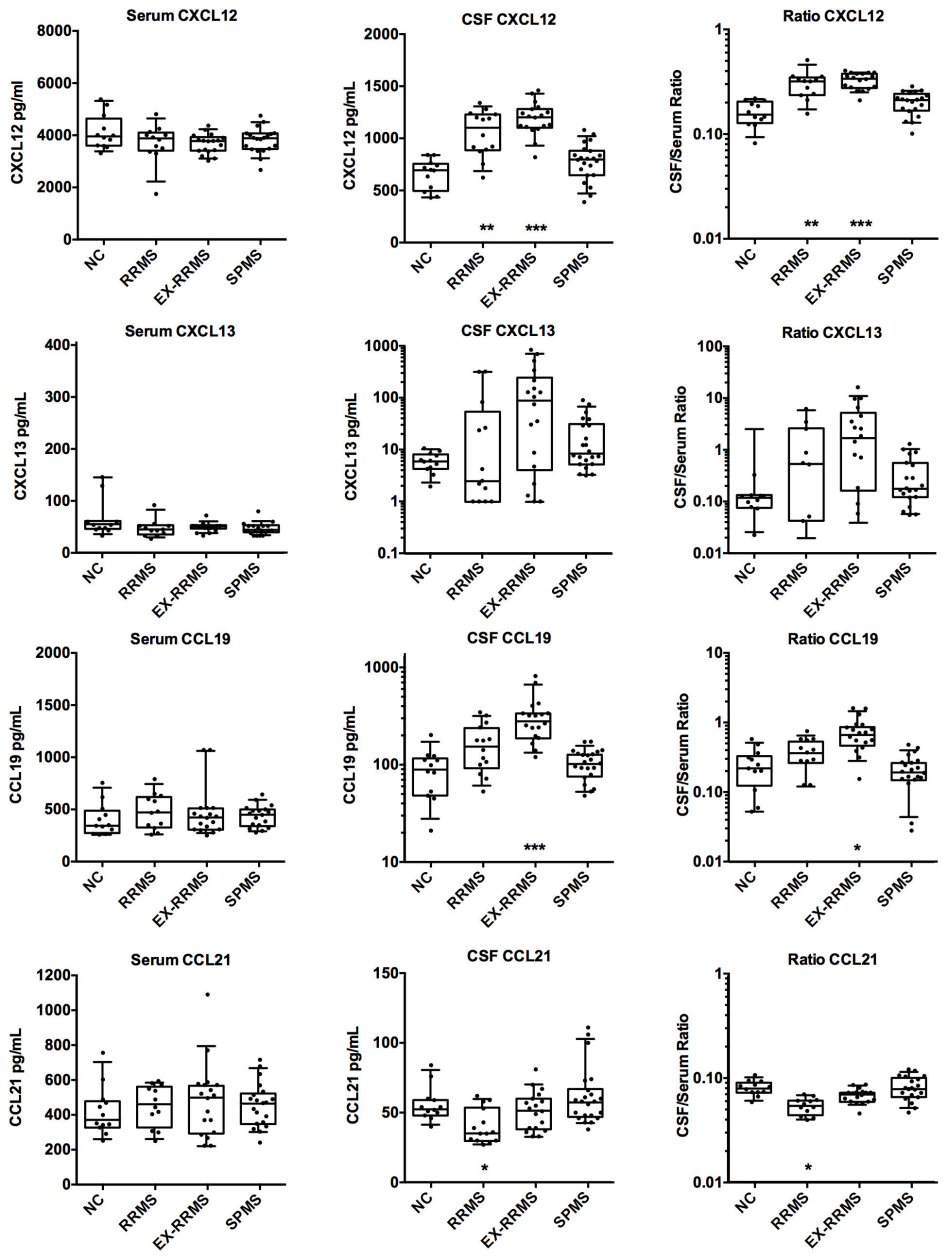
Comparison of the levels of the lymphoid chemokines CXCL12, CXCL13, CCL19 and CCL21 in serum and CSF. Chemokine concentrations are plotted with box and whiskers (10-90% range) overlaid on the scatter plots (each patient is a symbol). The CSF/serum ratio is presented on the right side. Data are shown for normal controls (NC), relapsing-remitting MS (RRMS), RRMS patients with acute exacerbations (EX-RRMS) and secondary progressive MS patients (SPMS). In those cases with EX-RRMS (9), SPMS (11) and RRMS (2) patients with second lumbar punctures, data from both samples are included. Statistical significance was assessed using only the baseline data and is indicated by asterisks at the bottom of each graph (ANOVA). Data at the lower limit of quantitation were excluded from the ratio plots.

 CSF samples from MS patients had elevated levels of CXCL12, CXCL13 and CCL19 when compared to normal controls; in contrast, CCL21 levels were either unchanged or dropped in RRMS patients (Figure 2). The decrease in CSF CCL21 levels is suggestive of increased consumption without increased production. As expected based on previous reports, EX-RRMS patients had the highest lymphoid chemokine levels followed by stable-RRMS and then SPMS patients. CXCL13 CSF levels were elevated in some but not all SPMS patients. Serum concentrations were not appreciably elevated in any group and hence the CSF/serum ratios for CXCL12, CXCL13 and CCL19 increased consistent with increased local CNS production. 

**Figure 3:**
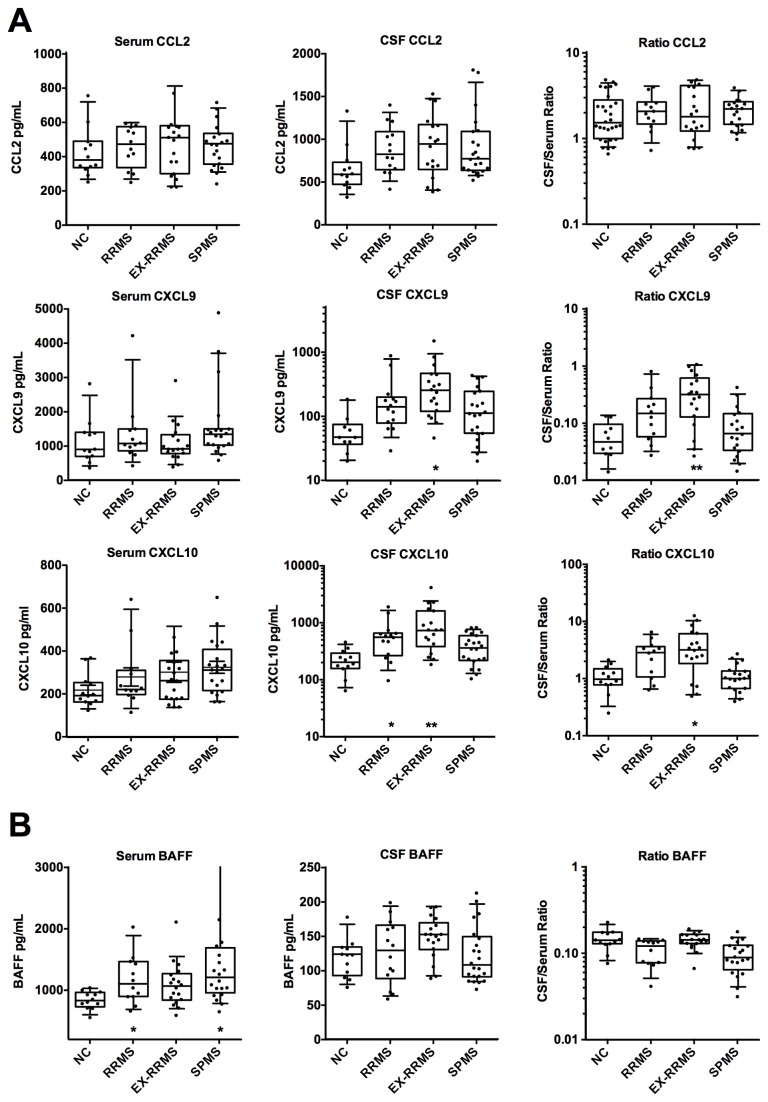
Comparison of the levels of the inflammatory chemokines in serum and CSF. (A) Concentrations of CXCL9, CXCL10, and CCL2 and (B) the cytokine BAFF are plotted as in Figure 2.

Using the inflammatory chemokine panel, MS patients were found to have elevated levels of CXCL9 and CXCL10 in the CSF (Figure 3). This effect was most prominent in the EX-RRMS group, but was also detectable in stable-RRMS (CXCL10), and trends were observed for SPMS patients. The elevated CXCL10 levels trended similarly to those reported earlier [35,36]. CXCL11 levels in CSF were generally too low for quantitation by the multiplex assay. CSF CCL2 levels were slightly elevated in MS patients, in contrast to a previous report where levels were decreased [36]. Serum levels of inflammatory chemokines were basically unchanged in both RRMS cohorts, yet the SPMS cohort had a trend towards elevated BAFF and CXCL10 values. 

**Figure 4:**
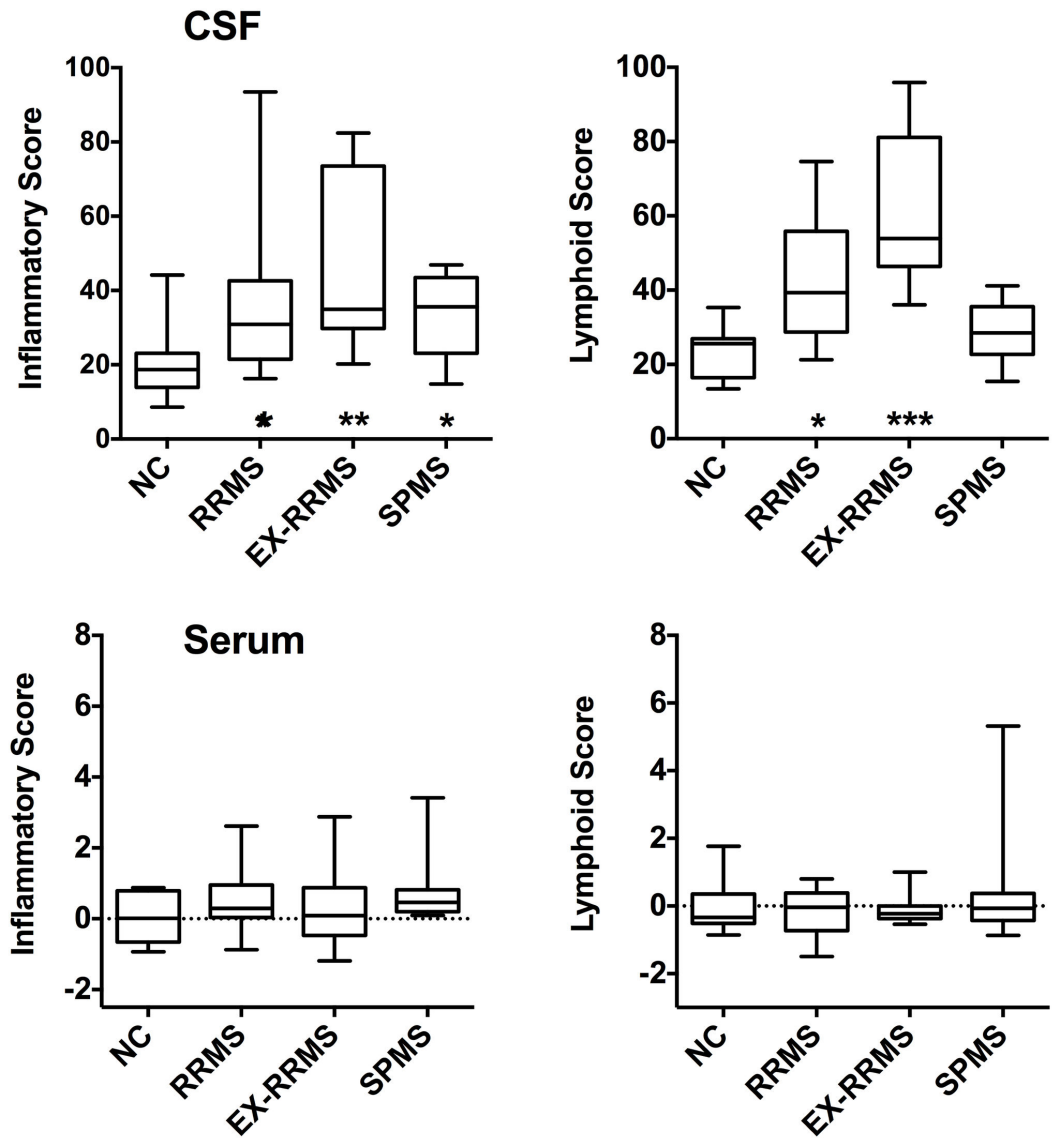
Composite scores for inflammatory (CCL2, CXCL9 and CXCL10) and lymphoid (CXCL12, CXCL13 and CCL19) chemokines. Baseline-only data are presented for both serum and CSF from NC, RRMS, EX-RRMS and SPMS patients. The composite score for the CSF data is derived from a normalized scale, while the serum data represent the mean of the z-values. Statistical significant is indicated by asterisks at the bottom of each graph (one-way ANOVA to NC).

**Figure 5:**
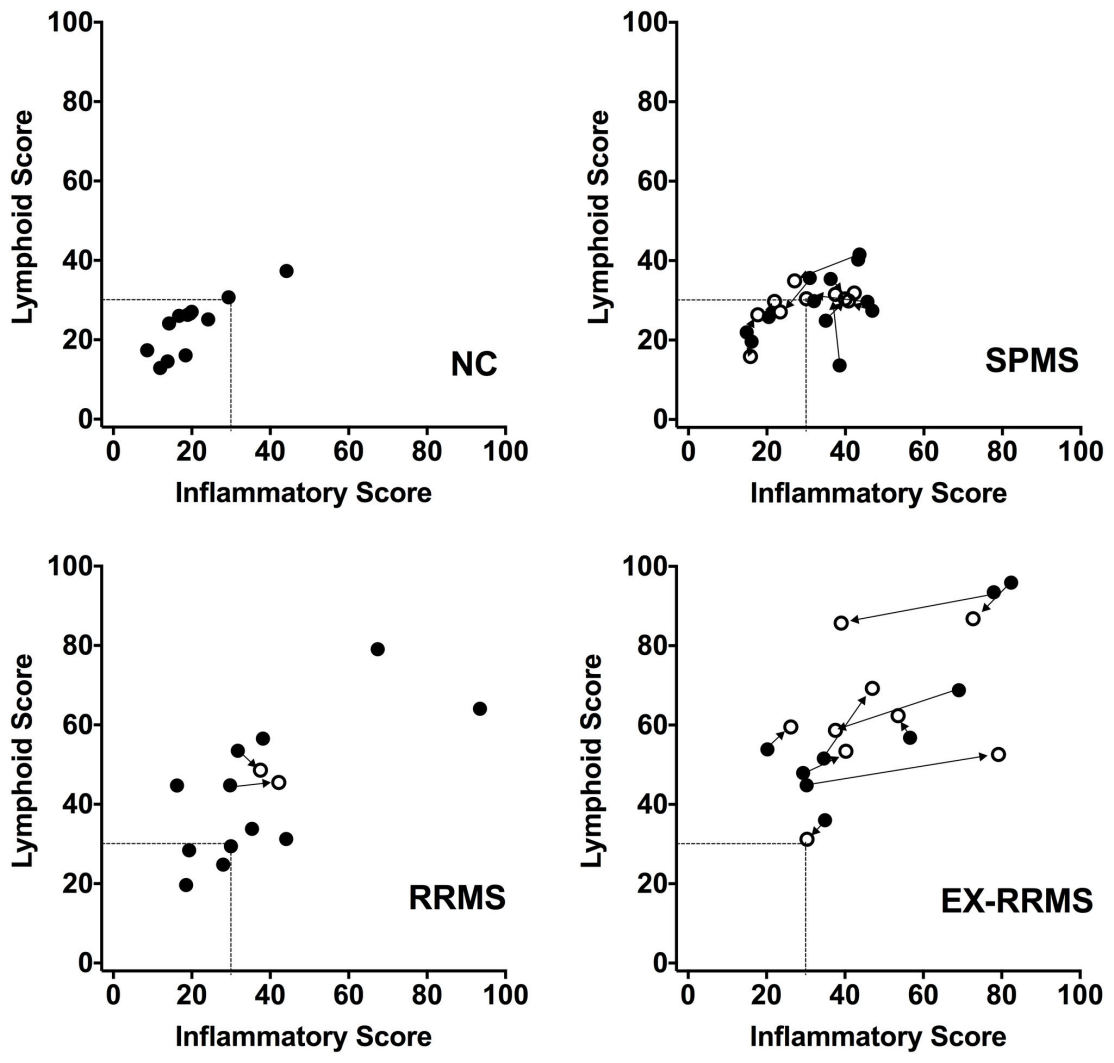
Relationship between the CSF lymphoid and inflammatory scores for MS patients and healthy normal controls. Solid circles show the baseline data while open circles are from the repeat lumbar puncture. Lines with arrow heads indicate paired samples from each patient (most MS subjects from the RRMS group did not have two lumbar punctures).

Given the parallel movement of several of these chemokines, we created a chemokine composite score for the lymphoid chemokines using the values for CXCL12, CXCL13 and CCL19. A second composite inflammatory chemokine score was derived from the CXCL9, CXCL10 and CCL2 values. Composite scores readily discriminated RRMS from normal controls (Figure 4). The inflammatory score for SPMS patients generally resembled that of RRMS patients, however, lymphoid scores tended to be lower in SPMS patients. When the relationship between the two scores was examined, RRMS patients in exacerbation appeared to have higher scores in both categories (Figure 5). Upon steroid treatment (arrows in Figure 5 show the change between the two CSF samples) the high inflammatory scores in three EX-RRMS patients experienced large shifts to the left, i.e. decreased inflammation, while two patients responded with increased inflammation by this definition. Two different combined inflammatory and lymphoid chemokine scores were investigated using CCL2, CXCL10 and CCL19 (score used successfully in lupus) and CXCL9, CXCL10, CXCL13 and CCL19 data. The inflammatory chemokine score still best discriminated between normal and SPMS.

Serum chemokine levels of MS patients were similar to those in normal controls, with trends for increased BAFF and CXCL10 levels in SPMS serum. Both BAFF and CXCL10 can be induced by interferon and indeed were elevated in a previous study of RRMS patients treated with IFN-ß that utilized this multiplex assay [37]. As 3 of 12 SPMS patients in this study were being treated with IFN-ß, the treatment itself may have driven these elevations. However, BAFF levels were not preferentially elevated in the IFN-ß treated patients although CXCL10 trended higher in this subset. A combined BAFF/CXCL10 serum score showed a significant difference between the normal and SPMS groups even when the IFN-ß treated patients were excluded (Figure S1 in File S1). IFN RNA signatures as defined originally in the blood of lupus patients have been noted in MS patients. Expression of blood and CSF RNA for IFN induced genes is currently being investigated to determine whether endogenous IFN could account for the serum BAFF/CXCL10 signature.

### Chemokine Levels and the Blood Brain Barrier

**Figure 6:**
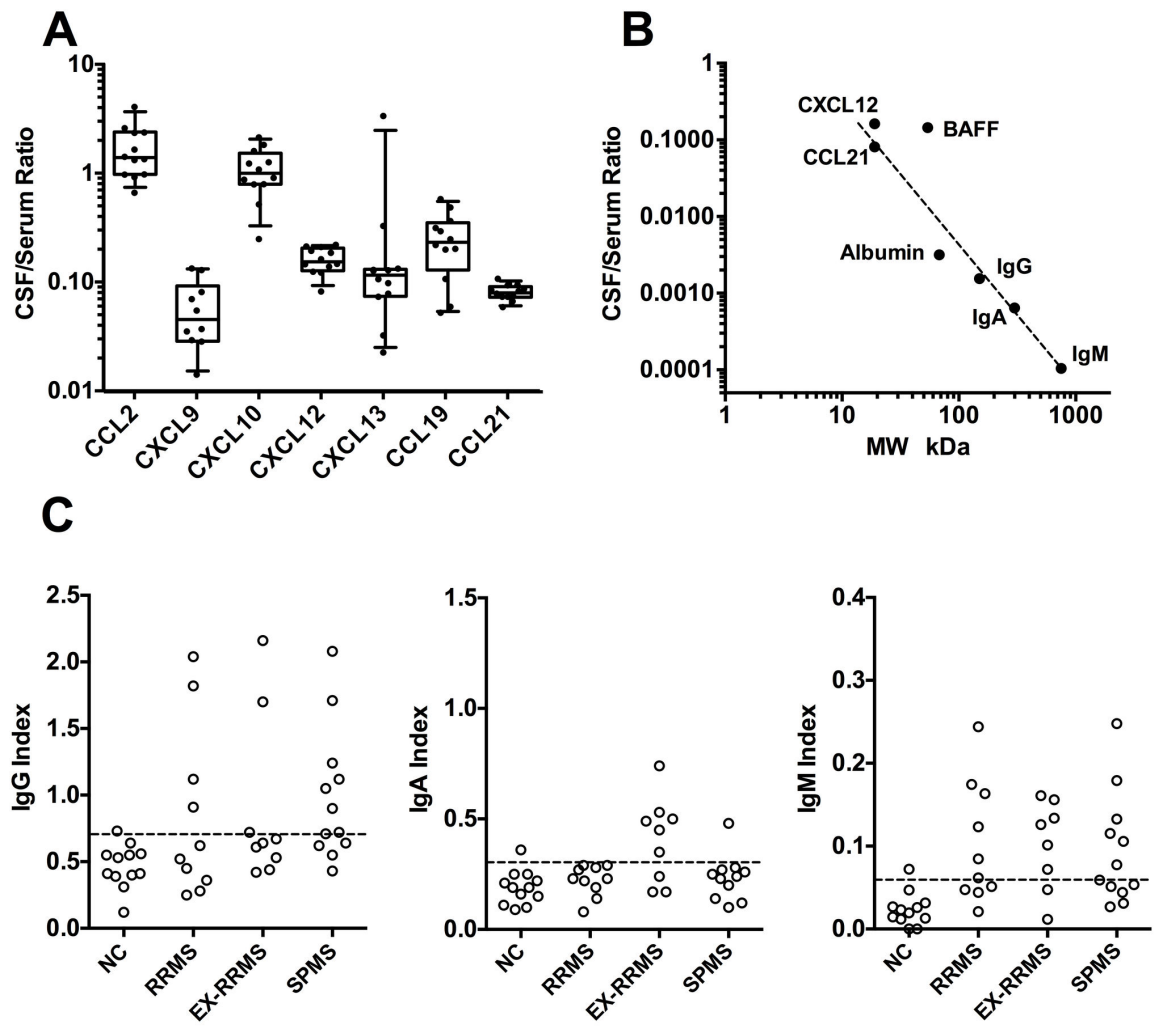
Ratio of CSF to serum chemokine levels in normal patients as well as the relationship to molecular size. A). Ratio of the CSF to serum chemokine concentrations for normal controls. B). Relationship between the molecular weight of the analyte and the CSF/serum ratio (only normal subjects). Only CCL21 and CXCL12 are plotted and CXCL9, CXCL13 and CCL19 would roughly overlay these values. Chemokines were assumed to be dimeric, BAFF trimeric, IgG dimeric, IgA tetrameric and IgM pentameric. Values above the line are consistent with local CNS production. C). Corresponding Ig indices for the different cohorts.

Analysis of the ratio of the CSF-to-serum levels of the various chemokines in healthy subjects indicated that except for CCL2 and CXCL10, there was 6-30 fold less chemokine in the CSF compared to the blood (Figure 6A). When these ratios for chemokines were compared to those of albumin, IgG, IgA and IgM in healthy subjects where no local CNS synthesis is expected, a reasonable relationship with the molecular size was observed (Figure 6B). Being small, chemokines are relatively permeable to the blood-CSF barrier. CCL19 and CCL21 have differing capacities to bind matrix component, yet their ratios were quite similar. This observation suggests that an increased chemokine ratio may reflect local CNS synthesis. Interestingly in normal controls the CCL2 and CXCL10 ratios differed substantially from the other chemokines with ratios around 1 suggesting local production in the CNS, although other more complex phenomena may be in play. BAFF is a glycosylated trimer of about 60 kDa approaching the size of albumin, albeit higher MW forms of BAFF have been described. BAFF deviated substantially from this molecular size relationship in normal controls also indicative of sources in the non-inflamed brain. 

The IgG and IgM indices were elevated in all MS cohorts, (Figure 6C), yet local synthesis of IgA may be associated with exacerbation possibly seen in an earlier analysis [23]. The IgG indices for all participants in this study were also analyzed according to Reiber (Figure S2 in File S1) and on this basis significant elevations were observed in some patients in all three MS cohorts [38]. Low-level blood contamination can affect the Ig indices but since CSF chemokines levels are within 5-100% of the serum values, they are unlikely to be altered by minor blood contamination. Of the 65 CSF samples for which RNA data were available, 5 showed some blood contamination with either clear RBC/neutrophil RNA signatures or RBC counts > 100 cells/µl as defined in the methods. The CSF samples with elevated Ig Indices did not correlate with those CSF samples that exhibited signs of blood contamination. 

### Correlations between Chemokine levels in the CSF

 Analysis of all the CSF data showed that in general the expression of the inflammatory and lymphoid chemokines scores correlated well using data from all patients (r = 0.72, baseline only). Among the lymphoid chemokines, similar patterns were seen with CXCL12, CXCL13, CCL19, while CCL21 was not coupled. When individual inflammatory and lymphoid chemokines were compared, a correlation was observed between CCL19 and CXCL10 or CXCL9 (r = 0.72, all data) (Figure S3 in File S1). The two inflammatory chemokines CXCL9 and CXCL10 tracked tightly with each other in the CSF (r = 0.92) and to a lesser extent in serum (r = 0.49). When CXCL13 levels were correlated with the lymphoid or inflammatory composite scores (Figure S4 in File S1), high levels of CXCL13 appear to correlate with either inflammation or lymphoid scores, but at levels below 20-30 pg/ml CXCL13, there was little correlation. Only 4 out 12 SPMS baseline CSF samples had elevated levels of CXCL13 relative to the normal controls. 

Previous analyses reported a correlation in MS between the Q_IgG_ and CSF CCL19 and CXCL13 levels [23,28,39]. This relationship was also examined in these cohorts and interestingly the correlation with CXCL13 was best with Q_IgM_ (r = 0.52) and was poorer with Q_IgA_ or Q_IgG_ (Figure S4 in File S1). CCL19 levels correlated somewhat with Q_IgG_ and Q_IgM_ (r = 0.45 and 0.46) and poorly with Q_IgA_ (r = 0.27) (data not shown). When each cohort was examined separately, CCL19 was decreased in SPMS despite retention of elevated Q_IgG_ values (Figure S5 in File S1). These data are consistent with the stable nature of the Q_IgG_ values as well as pointing to the potentially increased significance of CSF IgM [40]. 

### Longitudinal Analysis of CSF chemokines

**Figure 7:**
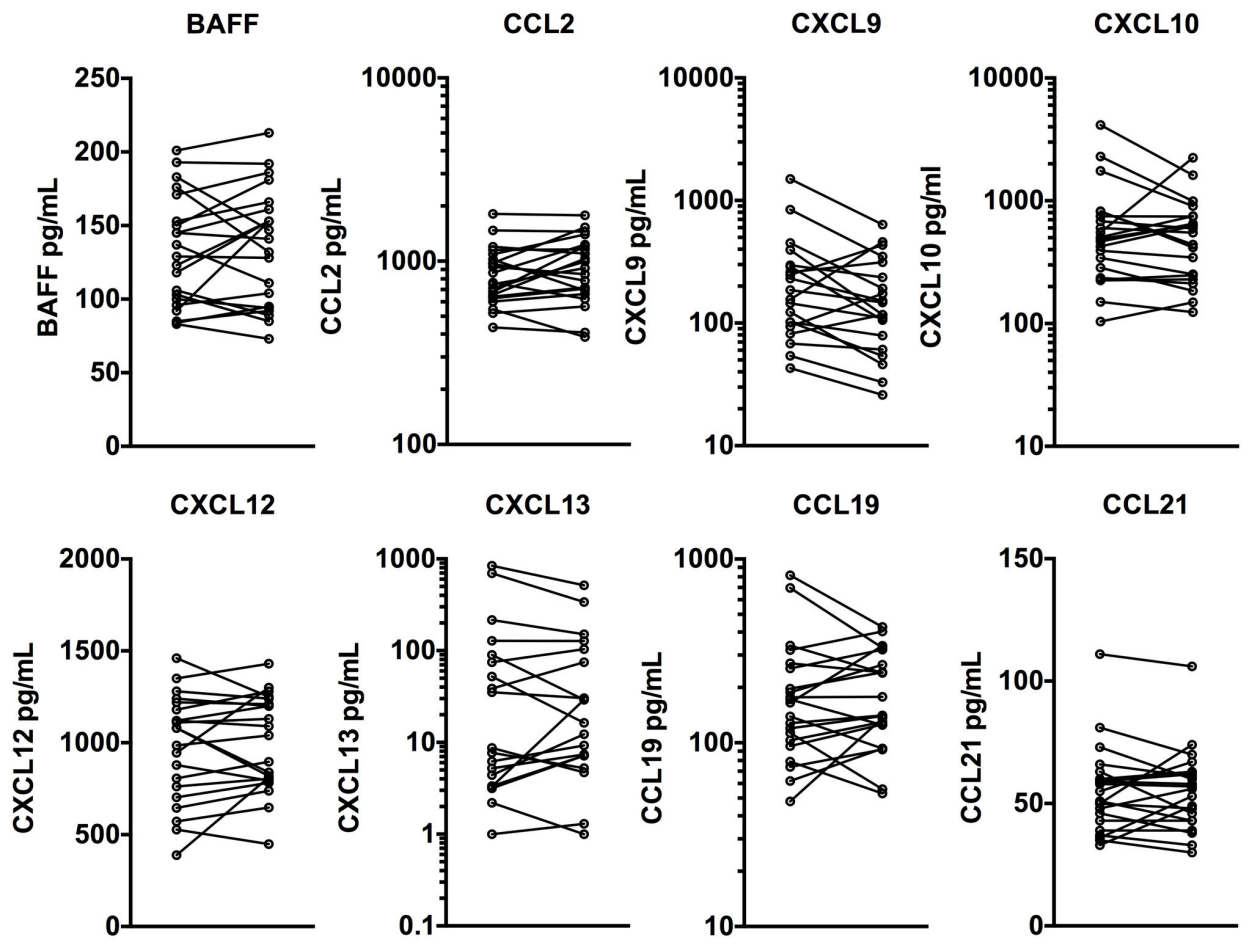
Trend analyses for each analyte for those individuals with two CSF samples. Both EX-RRMS and SPMS patients are grouped in the same graph.

In this study, 11 SPMS subjects had two lumbar punctures performed 1-18 months apart (average 337 days apart) and 9 RRMS subjects undergoing an exacerbation had a lumbar puncture and then received treatment with high dose IVMP. A second lumbar puncture was performed one month after treatment. In general, the chemokine levels were stable over time (Figure 7). In several subjects with a 50-100% change in the chemokine level, a similar upward or downward trend was noted in multiple analytes. The administration of high dose IVMP to EX-RRMS patients had some impact in a subset of patients on the CSF chemokine levels, e.g. CCL19 levels as well as the chemokine scores decreased in 3/9 patients one-month post IVMP (there was no prednisone taper), while the remainder were unchanged or increased (data not shown and Figure 5). Little change in CXCL10 levels following IVMP treatment was noted earlier and this observation is in general agreement with our data [35,36]. The data indicate that CSF chemokine levels in SPMS patients remain relatively stable over intervals of up to 1.5 years.

## Discussion

The goal of this study was to explore measurement and analysis methods to compare the levels of chemokines in both the serum and CSF in the relapsing and progressive phases of MS. Specifically, we wished to develop more robust metrics that could serve as surrogate indicators for meningeal/cortical inflammation in SPMS in the context of clinical trials. The composite chemokine scores defined here are promising and can be applied to future studies. In these small cohorts of patients with relatively advanced disease, neither group of chemokines tracked preferentially with one particular disease stage. Heterogeneity in disease status, medication usage, advanced disease or substantial contributions of subclinical disease may have masked potential differences in the chemokine patterns and larger studies would be required to elucidate this important question. 

This study confirms and extends previous observations demonstrating that both inflammatory and lymphoid chemokines are elevated in the CSF in RRMS especially during exacerbation [13,16,22,23,28,34,39,41,42]. While the magnitude of the chemokine response varied by disease stage, the relative proportion of inflammatory or lymphoid chemokines did not vary substantially. In SPMS, the inflammatory chemokine levels were similar to clinically stable RRMS patients, while lymphoid chemokine levels were slightly lower. The median interval from conversion to SPMS was 8.7 years in this SPMS cohort and hence the similar nature of stable RRMS and SPMS is not due to recent conversions. Older MS patients, i.e. greater than 54 years of age, had in general lower CXCL13 levels in the CSF, yet our cohort of SPMS patients with a mean age of 46 was similar to the stable RRMS group [27]. Despite the readily detectable CNS inflammation, there was little change in the serum levels of these chemokines in any of the MS groups we studied. 

Barring the small size of this feasibility study, there are several scenarios that could explain the lack of qualitative differences in the CSF chemokine signatures between the stable RRMS and SPMS subjects we studied. First, the general notion that lymphoid chemokines are preferentially associated with meningeal inflammation as opposed to CNS inflammation in general in MS could be erroneous. Second, the contribution of the progressive cortical component of the disease in SPMS may be small relative to the classical white matter component. Third, the nature of the immunology/inflammation could be very similar at these two stages at least as reflected in the CSF chemokine milieu. Given the presence of substantial cortical inflammation early in the disease process i.e. at the CIS stage, multiple pathologies may already be present during the relapsing phase of the disease making the differentiation of RRMS from SPMS by CSF chemokine signatures not possible [5]. 

There were some surprises in this analysis. CCL19 and CCL21 are in theory both lymphoid, yet tracked differently in this study. Both chemokines interact similarly with CCR7 to promote naïve and central memory T cell trafficking. CCL21 is efficiently immobilized onto matrix and in this state can drive T cell crawling along reticular networks or lymphocyte entry into lymphatic compartments. In contrast CCL19 remains more soluble and therefore may report more accurately on relative levels of immune activity. The apparent increased consumption of CCL21 in the face of increased CSF CCL19 could be consistent with these differing biochemical properties. CXCL12 is produced by the vasculature and astrocytes and is key for retention of lymphocytes within the perivascular compartment [43]. Loss of CXCL12 in rodent models allows lymphocytes to leave the perivascular space and enter the parenchyma. As such, CXCL12 is a lymphoid chemokine that should be present in the CSF, yet varying results have been obtained regarding its presence in CSF [16]. Our multiplexed data are effectively identical with one of the earlier reports [39]. The impact of elevated levels of CXCL12 on the disease process is unclear. Another surprising finding was the suggestion of a BAFF/CXCL10 chemokine signature in the blood that was more distinctive in SPMS and RRMS compared to NC or EX-RRMS patients. IFN-induced RNA signatures have been noted in MS patients [44–46] and blood levels of both proteins are increased following IFN administration in MS patients [37,47]. The BAFF/CXCL10 elevation shown here may reflect endogenous activation of the IFN axis in these SPMS patients. In general, there have been few robust blood indicators of peripheral lymphoid involvement in MS [17,48]. This result is in stark contrast to other autoimmune diseases. 

CXCL13 levels in CSF have been widely explored and they have the potential to reflect B cell involvement in MS. Indeed, both total cellularity and B cell content in the CSF correlated well with CXCL13 concentrations [16,23]. In this light, the levels of CXCL13 correlated well with the Q_IgM_ values, an observation that could be consistent with the recent suggestion that IgM oligoclonal bands may track with brain atrophy and lesion load [40,49]. We found that CXCL13 CSF levels are generally increased in MS, yet only about a third of the SPMS patients in this cohort had elevated levels. Despite the use of a high sensitivity CXCL13 iPCR to improve quantitation of low levels in CSF, CSF CXCL13 may be a challenging metric for clinical studies during the progressive phase. 

The CSF to serum ratios in the normal subjects for these chemokines, BAFF, albumin, IgG, IgA and IgM were roughly consistent with a simple size dependence for the blood-CSF barrier. However, CCL2 and CXCL10 (but not CXCL9) as well as BAFF had higher CSF/serum ratios when compared to the other chemokines, suggesting intrathecal production in the normal CNS. The production of substantial amounts of BAFF in the normal CNS has been well documented [50]. The CXCL12 and CXCL13 CSF/serum ratios were roughly similar to those reported in a recent analysis of CNS lymphoma [51]. Also consistent with our data, a high ratio of 5 was reported for CXCL10 in RRMS patients [35]. Chemokines can bind to matrix components and this aspect could confuse this analysis, yet both CCL19 and CCL21 had roughly similar CSF/serum ratios despite having differing matrix binding C-terminal regions. CCL2 is clearly induced in the injured or inflamed brain, especially in astrocytes, yet the results from quantitation of CCL2 levels in the normal rodent brain are mixed ranging from no detectable RNA to detectable RNA and protein [52]. Local chemokine production by myeloid lineage cells lining the meningeal compartments could also bias this ratio. CCL19 was reportedly present in the normal CNS along with its RNA whereas there was no detection of CCL21 [28]. In this study, the CSF to serum ratio for CCL19 in healthy controls was slightly higher than CCL21, CXCL13 or CXCL9 and therefore our data could be consistent with some local CNS production of CCL19. 

In summary, we developed robust methods for the measurement and analysis of inflammatory and lymphoid chemokines in the CSF of MS patients. Results of a pilot study in a small number of subjects from a single clinic failed to reveal major differences between stable RRMS and SPMS. Composite “chemokine scores” may form the basis for more usable clinical biomarkers and patient stratification in stable RRMS and SPMS subjects and warrant further evaluation in larger cohorts of patients and possibly in clinical trials. 

## Supporting Information

File S1 Figures S1-S5.
(PDF)
