# Supplementary material for: Feasibility of the Use of Combinatorial Chemokine Arrays to Study Blood and CSF in Multiple Sclerosis
Source: PLoS One. 2013 Nov 20;8(11):e81007. doi: 10.1371/journal.pone.0081007 (PMC3835751; doi:10.1371/journal.pone.0081007)
Supplement: File S1 — Figures S1-S5. (PDF) [file pone.0081007.s001.pdf]

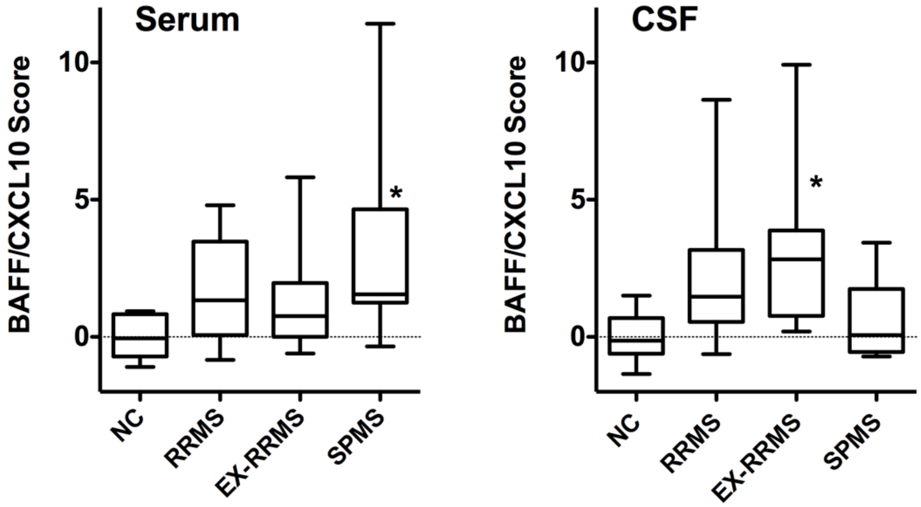

Supplemental Figure S1:

Composite BAFF-CXCL10 score is elevated in the serum of SPMS patients. The BAFF-CXCL10 signature score represents the average of the z-scores for the two analytes and is elevated primarily in in the serum from SPMS and the CSF of RRMS and EX-RRMS patients. Data include all baseline data. Asterisk indicated $p < 0.05$ for comparison to NC, result of ANOVA between all pairs. An analysis (not shown) that excluded patients who had been receiving interferon-β treatment (total of 7 patients, 1 EX-RRMS, 3 RRMS and 3 SPMS) yielded the same result.

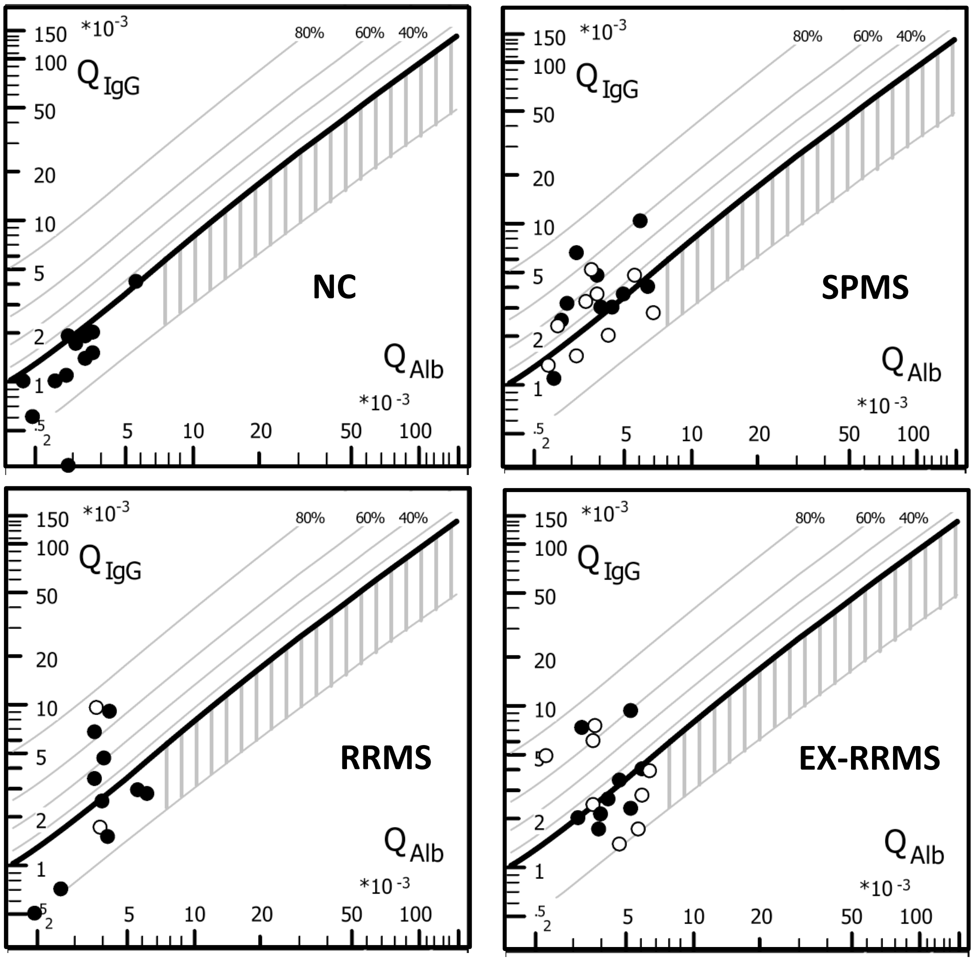

Supplemental Figure S2:

Analysis of IgG Indices in different cohorts. The method of Reiber was used to plot the IgG index for each patient. Solid symbols indicate baseline CSF while open symbols represent the second CSF sampling. Contour lines represent the confidence levels for abnormal values.

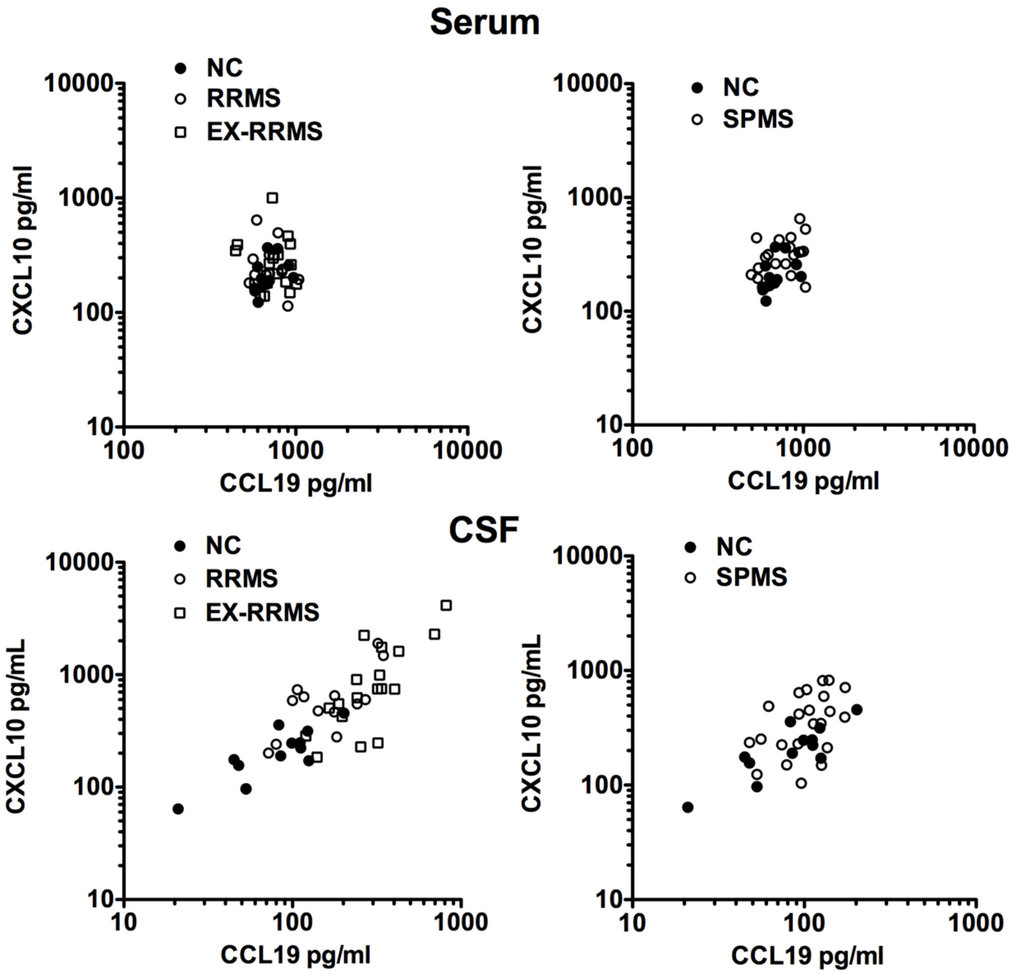

Supplemental Figure S3:

Relationship between CSF levels of CXCL10 (inflammatory chemokine) and CCL19 (lymphoid chemokine). CSF concentrations are plotted for each of the baseline and repeat CSF samples.

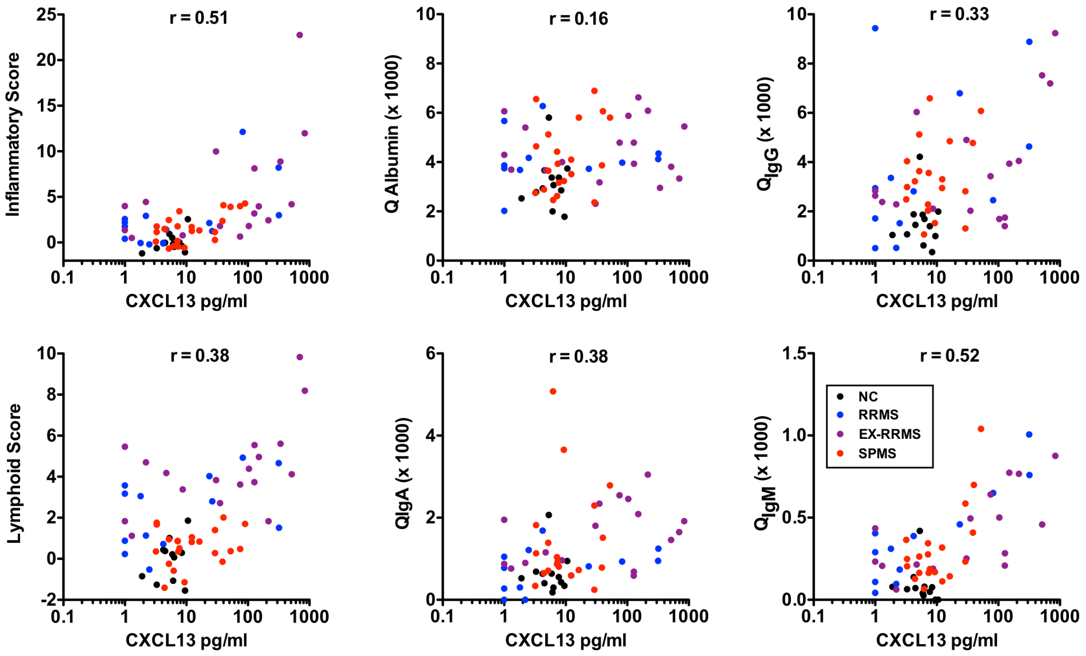

Supplemental Figure S4:

Relationships between CSF Ig Q ratios and various chemokine measurements. CSF CXCL13 levels are compared to both the inflammatory and lymphoid chemokine scores and CSF/serum Q ratios for IgG, IgA, IgM and albumin. Each point represents a sample and both baseline and repeat samples are included (Spearman coefficients are shown).

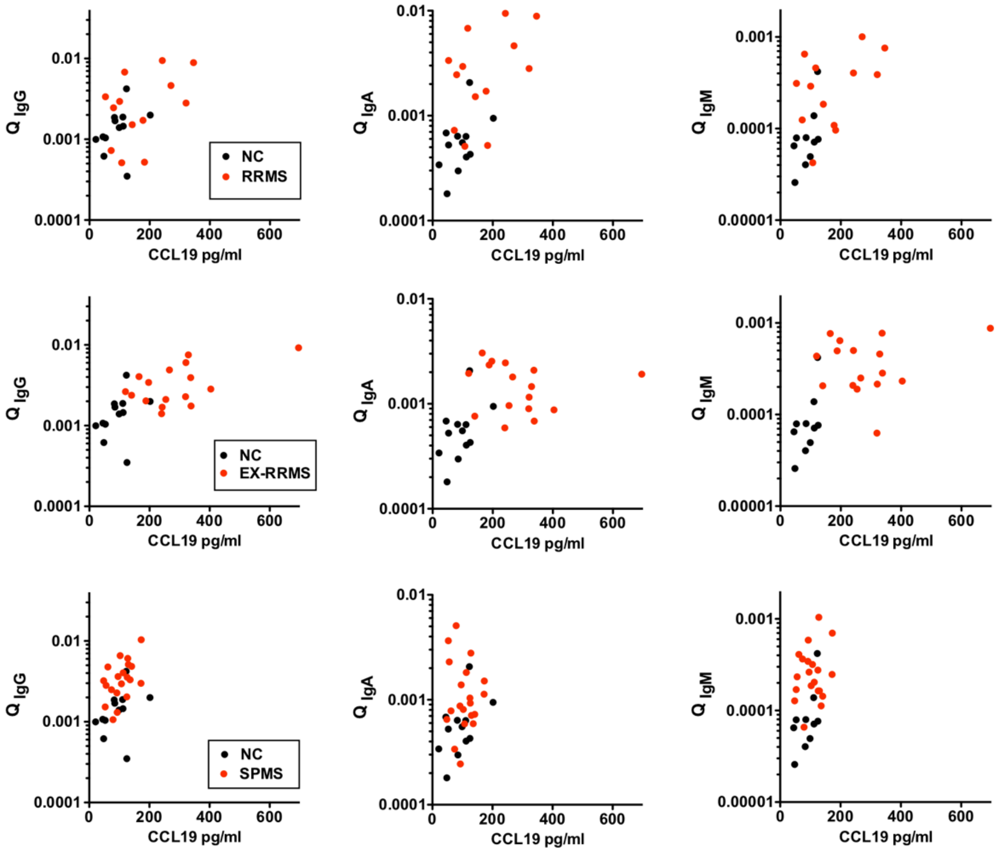

Supplemental Figure S5:

Relationships between CSF Ig Q ratios and various chemokine measurements. Relationship CSF CCL19 levels between the Q values for IgG, IgA and IgM for each cohort.
